# A Case of a Patient Who Is Diagnosed with Mild Acquired Hemophilia A after Tooth Extraction Died of Acute Subdural Hematoma due to Head Injury

**DOI:** 10.1155/2018/7185263

**Published:** 2018-12-09

**Authors:** Tomohisa Kitamura, Tsuyoshi Sato, Eiji Ikami, Yosuke Fukushima, Tetsuya Yoda

**Affiliations:** ^1^Department of Oral and Maxillofacial Surgery, Saitama Medical University, 38 Moro-hongou, Moroyama-machi, Iruma-gun, Saitama 350-0495, Japan; ^2^Department of Maxillofacial Surgery, Tokyo Medical and Dental University, Tokyo, Japan

## Abstract

**Background:**

Acquired hemophilia A (AHA) is a rare disorder which results from the presence of autoantibodies against blood coagulation factor VIII. The initial diagnosis is based on the detection of an isolated prolongation of the activated partial thromboplastin time (aPTT) with negative personal and family history of bleeding disorder. Definitive diagnosis is the identification of reduced FVIII levels with evidence of FVIII neutralizing activity.

**Case report:**

We report a case of a 93-year-old female who was diagnosed as AHA after tooth extraction at her home clinic. Prolongation of aPTT and a reduction in factor VIII activity levels were observed with the presence of factor VIII inhibitor. AHA condition is mild. However, acute subdural hematoma of this patient occurred due to an unexpected accident in our hospital. Hematoma was gradually increased and the patient died 13 days after admission.

**Discussion:**

Although AHA is mild, intracranial bleeding is a life-threatening condition. We also should pay attention to the presence of AHA patients when we extract teeth.

## 1. Introduction

Acquired hemophilia A (AHA) is a rare hemorrhagic disease caused by autoantibodies against blood coagulation factor VIII. The incidence of AHA is 14.7 per million per year in the elderly over 85 years, and more than 80% of AHA patients is 65 years or older [1]. On the other hand, 25 cases per 16 years has been reported at a single center in Japan [2]. The initial diagnosis is based on the detection of an isolated prolongation of the activated partial thromboplastin time (aPTT) with negative personal and family history of bleeding disorder. Definitive diagnosis is the identification of reduced FVIII levels with evidence of FVIII neutralizing activity. In this report, we describe a case of AHA who was diagnosed after tooth extraction.

## 2. Case Report

**Figure 1:**
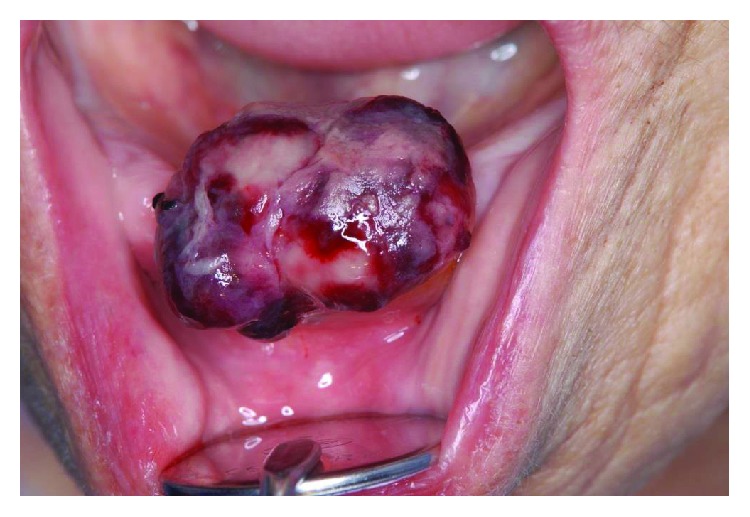
The hematocele on the gingiva area (after tooth 32 extraction). Photo of a mass on gingiva.

A 93-year-old female patient with hemorrhage after tooth extraction (tooth 32) was referred to our hospital on Apr 2014. Eight days before the transfer to the hospital, her teeth were extracted by a primary dentist. Her gingival hemorrhage recession was at the same place as tooth extraction. She had no past medical history and also she took no medications. Blood clot in the socket has been increased 6 days after extraction, resulting in difficulty to have meals. On oral examination, blood clot formed a pedunculated mass on gingiva, and the size of mass was 22 mm × 15 mm × 7 mm (Figure 1).

**Figure 2:**
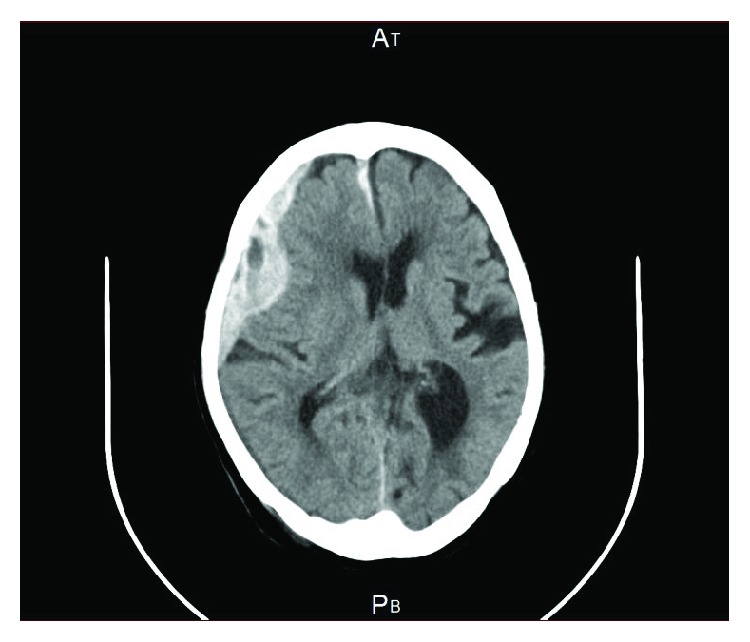
Magnetic resonance imaging of the horizontal section at the frontal lobe of the cerebrum.

As laboratory data showed that a prolonged aPTT was beyond normal range (70.7 sec), hemorrhagic diathesis was suspected. We thus consulted with a hematologist. On the 4th day after admission to hospital, aPTT cross-mixing test revealed a reduction in factor VIII (FVIII) activity levels (9%). Other hematological data including platelet count, prothrombin time (PT), and fibrinogen degradation products (FDP) were not out of the reference values. And any autoantibodies such as antinuclear antibody were not detected. Then AHA was suspected. On the same day, a nurse found that she was lying beside the bed at night. Since she complained headache, we suspected that she fell off the bed but no one knows what happened. We considered that falling form bed was one of the trigger of her subdural hemorrhage. Diffusion magnetic resonance imaging showed a high-intensity area in the frontal lobe of the cerebrum (Figure 2), suggesting acute subdural hematoma. Immediately, the patient was transferred to the emergency department. On the next day, the Glasgow Coma Scale was E4V4M6. A high titer of factor VIII inhibitor (7 units/ml) confirmed the diagnosis of AHA. We discussed with a hematologist whether to use eptacog alpha (Novoseven ®), but the hematologist did not recommend to use such drug because of the patient's age and expected prognosis. The hematologist also did not recommend prednisolone therapy, and fresh frozen plasma infusion was performed. aPTT and FVIII activity levels were significantly improved 52.9 sec and 35%, respectively. Emergency physician decided not to perform surgery because of high risk due to AHA and advanced age. Hematoma was gradually increased on CT examination and decreased level of consciousness (E1V1M3). Then the patient died 13 days after admission.

## 3. Discussion

An isolated aPTT prolongation suggests a deficiency or inhibitor of one or more of the intrinsic pathway clotting factors including prekallikrein, high-molecular-weight kininogen, and factors XII, XI, IX, and VIII. From the viewpoint of clinical medicine, two conflicting disease categories, such as hemorrhagic diseases and thrombotic diseases, should be considered. Hemorrhagic disease is AHA, and thrombotic disease is antiphospholipid syndrome with positive lupus anticoagulant [3]. AHA exhibits reduction of FVIII levels. Unlike congenital hemophilia A, the clinical severity is irrelevant to FVIII levels in AHA. AHA is diagnosed by the presence of FVIII inhibitors.

To detect AHA, it is needed to check blood test including APTT and PT. We recommend to administer blood test before tooth extraction to avoid hemorrhage. We also recommend dentists to cooperate with physician before invasive treatment and check the blood conditions.

The hematologist did not use prednisolone for the treatment of AHA because they were apprehensive of infection due to advanced age. In this case, FFP infusion improved aPTT and FVIII activity, suggesting mild AHA. However, AHA patients are at high risk of death. Indeed, unfortunate accident has happened in our case. Although AHA is mild, intracranial bleeding is a life-threatening condition.

The critical cause of AHA is autoantibodies against autologous factor VIII. It is said that AHA is associated with autoimmune disease, malignancy, pregnancy, infection, aging, or certain medications. This case related only aging.

We range 5 papers of AHA cases in the region of dentistry over references in NCBI Reference Sequences [4–8]. Only 3 papers concerning about tooth extraction of AHA patients have been reported [4–6]. Although there are a few reports in dentistry, it is conjectured that general dentists encounter AHA patients more often than we expected. As they did not write papers for case reports usually, we cannot catch those cases. Tooth extraction is the most common surgical procedure among dental treatments for general dentists. As they do not check the patients' laboratory data routinely, postoperative hemorrhage may be an important sign. Dentists should pay attention to the presence of AHA patients.
